# Understanding the interactions of genotype with environment and management (G×E×M) to enhance maize productivity in Conservation Agriculture systems of Malawi

**DOI:** 10.1371/journal.pone.0298009

**Published:** 2024-04-29

**Authors:** Blessing Mhlanga, Mphatso Gama, Richard Museka, Christian Thierfelder

**Affiliations:** 1 International Maize & Wheat Improvement Centre (CIMMYT) -Southern African Regional Office, Harare, Zimbabwe; 2 Machinga Agriculture Development Division, Liwonde, Malawi; 3 Total LandCare Malawi, Lilongwe, Malawi; Nepal Agricultural Research Council, NEPAL

## Abstract

Climatic variability and soil fertility decline present a fundamental challenge for smallholder farmers to determine the optimum management practices in the production of maize. Optimizing genotype (G) and management (M) of maize under different environmental conditions (E) and their interactions are essential for enhancing maize productivity in the smallholder sector of Malawi where maize is the main staple food. Here, we evaluated over seven seasons, the performance of four commercial maize genotypes [including hybrids and one open pollinated variety (OPV)] managed under different Conservation Agriculture (CA) and conventional practices (CP) across on-farm communities of central and southern Malawi. Our results revealed significant G×E and E×M interactions and showed that hybrids such as DKC 80–53 and PAN 53 outyielded the other hybrid and the OPV in most of the environments while the OPV ZM523 had greater yields in environments with above-average rainfall and shorter in-season dry spells. These environments received a maximum of 1250 mm to 1500 mm of rainfall and yet the long-term averages were 855 mm and 1248 mm, respectively. Despite yielding lower, the OPV ZM523 also exhibited higher yield stability across environments compared to the hybrid MH 30, possibly due to its resilience to drought, heat stress, and low soil fertility conditions which are often prevalent in the target communities. Conservation Agriculture-based practices outyielded CP across the genotypes and environments. However, amongst the CA-based systems, intercropping of maize with pigeonpea [*Cajanus cajan* (L.) Millsp] and cowpea (*Vigna unguiculata* Walp.) performed less than monocropping maize and then rotating it with a legume probably due to competition for moisture between the main and the companion crops in the intercrop. The key findings of this study suggest the need to optimize varietal and management options for particular environments to maximize maize productivity in Malawi. This means that smallholder farmers in Malawi should adopt hybrids and CA-based systems for enhanced yields but could also consider OPVs where the climate is highly variable. Further rigorous analysis that includes more abiotic stress factors is recommended for a better understanding of yield response.

## 1. Introduction

Global food demand is increasing with the increasing population, and this has resulted in food shortages especially in the smallholder farming sector of southern Africa [1]. In Malawi, maize [*Zea mays* L.] is the staple food, the most important food security crop, and one of the main sources of both income and calories for smallholder farmers [2, 3]. Maize is grown in Malawi on over 75% of the arable land area and accounts for more than 50% of the caloric intake [4, 5]. However, maize yields are low as compared to global standards and this is due to many factors which include increasingly variable climate, poor or untimely management, and inadequate amounts of inputs, amongst others. In sub-Saharan Africa, increases in maize production are more associated with increasing cultivated area than maize yield improvement [6]. This calls for strategies that increase food production while being resilient to the effects of variable climate to meet food demands from an increasing population [7].

One of the ways to close this yield gap is adopting improved elite climate-resilient maize cultivars (with superior performance based on evaluation against other lines) and benefit from the significant genetic gains from breeding programs by different organizations such as the International Maize & Wheat Improvement Centre (CIMMYT) [8, 9]. Improved maize varieties (both hybrids and open-pollinated) are more adapted to the local conditions of southern Africa as they are bred to be more tolerant to key abiotic and biotic stresses using an extensive phenotyping, screening and on-farm testing system [10, 11]. The resultant varieties are more tolerant to different stress elements such as water stress; low nitrogen; lodging; pests; and diseases, amongst others. In other regions such as China and the USA, yields have tripled in the last 50 years, and this has been partly the result of rapid breeding cycles, effective selection, genetically modified organisms, rapid varietal turnover, and input use [12, 13]. Cairns and Prasanna [14] have shown that breeding programs have benefitted about 53 million people in sub-Saharan Africa, and this is a significant achievement considering the vulnerability of the smallholder cropping systems in this region.

Despite their high production potential, adapting improved genotypes to farmer environments continues to be challenging due to the complex interactions of genotypes and environments which have also complicated breeding efforts [15]. Climatic factors such as temperature and rainfall have a significant influence on the performance of maize cultivars, and these are highly variable in southern Africa. A study by Cairns et al. [8] forecasted delays in the onset of growing seasons in southern Africa coupled with early cessation of rains and increases in temperatures. These factors further exacerbate the challenges of breeding efforts and the adaptation of maize cultivars to smallholder conditions. Genotypic traits such as days to maturity also determine maize response to the environment, with early maturing cultivars being reported to be better adapted to unfavourable environments compared to extra early cultivars [8]. Besides understanding the genotype by environment interaction, agronomic management of crops is crucial as this is closely related to agroecosystem services and outputs. Hence, it is important to manage crops judiciously to benefit from the genetic potential of the crops while also reaping the additional benefits from improved and more climate-smart and resilient agri-food systems [7].

One of the cropping systems that has shown potential to increase crop productivity in southern Africa is Conservation Agriculture (CA) [16]. Conservation Agriculture is based on three principles: minimum soil disturbance, crop diversification, and permanent soil cover using organic material [17] amongst complementary good agriculture practices needed for it to thrive [18]. Conservation Agriculture has been shown in many studies to enhance crop yields and improve other agronomic, economic, and biological aspects (for more detailed studies please review [19–21]). Across southern Africa, CA practices are carried out in different forms, meaning that farmers may use different combinations of the CA principles and tools which may result in variable yield responses across the region [22].

Most of the studies done in southern Africa usually include one genotype of maize as the test crop. For example, multilocational studies by Mhlanga et al. [22] and Setimela et al. [23] tested the effects of different CA systems across southern Africa and in these studies, only one genotype was used as a test crop although they varied depending on agroecology. Another multilocation study by Thierfelder et al. [16] tested the effects of different CA practices in different environments but for each environment, only one genotype was used as the test crop. Limiting to these studies is the use of a single genotype in each environment depending on local recommendations from extension and private seed companies. Such an approach does not capture the genetic gain from breeding programs and also limits the potential of the interaction of locally available genotypes with environments.

Due to the socioeconomic importance of maize in Malawi, some farmers usually associate maize with life, as a source of livelihood [2], and periods of crop failure often lead to major disturbances in political stability. Considering that many genotypes are being released by different breeding programs and are available to farmers and that farmers are implementing different CA practices in different environments in Malawi, it is important to assess how these genotypes perform under different management practices in different environments. Optimizing this relationship between genotype and management practices is of utmost importance.

In Malawi, different genotypes have been bred for biotic and abiotic stress tolerance and these are available on the market from different seed houses. Farmers in Malawi are usually not well informed on the suitability of these different genotypes for their context hence the need for informed analyses that assist in making decisions. The aim of this study was therefore to assess the performance of the different genotypes available on the Malawian market (which include hybrids and an open-pollinated variety (OPV)) managed under different CA practices and conventional ridge tillage across 10 on-farm communities of central and southern Malawi. In this study, we hypothesized that: (i) hybrids yield more than OPVs across different environments, (ii) maize yield response is determined by a G×E×M effect and higher yields are obtained when hybrids are adopted in CA-based systems under variable environments, (iii) CA-based systems yield more than conventional practices across different environments, (iv) in response to its vigour and better adaptability, the OPV will result in more stable yields as compared to hybrids across these environments, and (v) hybrids and CA-based systems are less sensitive to seasonal precipitation and temperature variability across different environments. We acknowledge that precipitation and temperature are not the only important determining factors but more abiotic factors have been discussed in detail by Komarek et al. [24]. The information generated in this study will assist breeders in the region at large and smallholder farmers in Malawi in particular in capturing the potential of cropping practices to achieve greater and more reliable yields based on their specific contexts. Furthermore, the information will assist farmers who can invest in new seed to make use of genetic gains thus, maximizing the return on their investment.

## 2. Materials and methods

### 2.1. Description of the on-farm study sites

**Fig 1:**
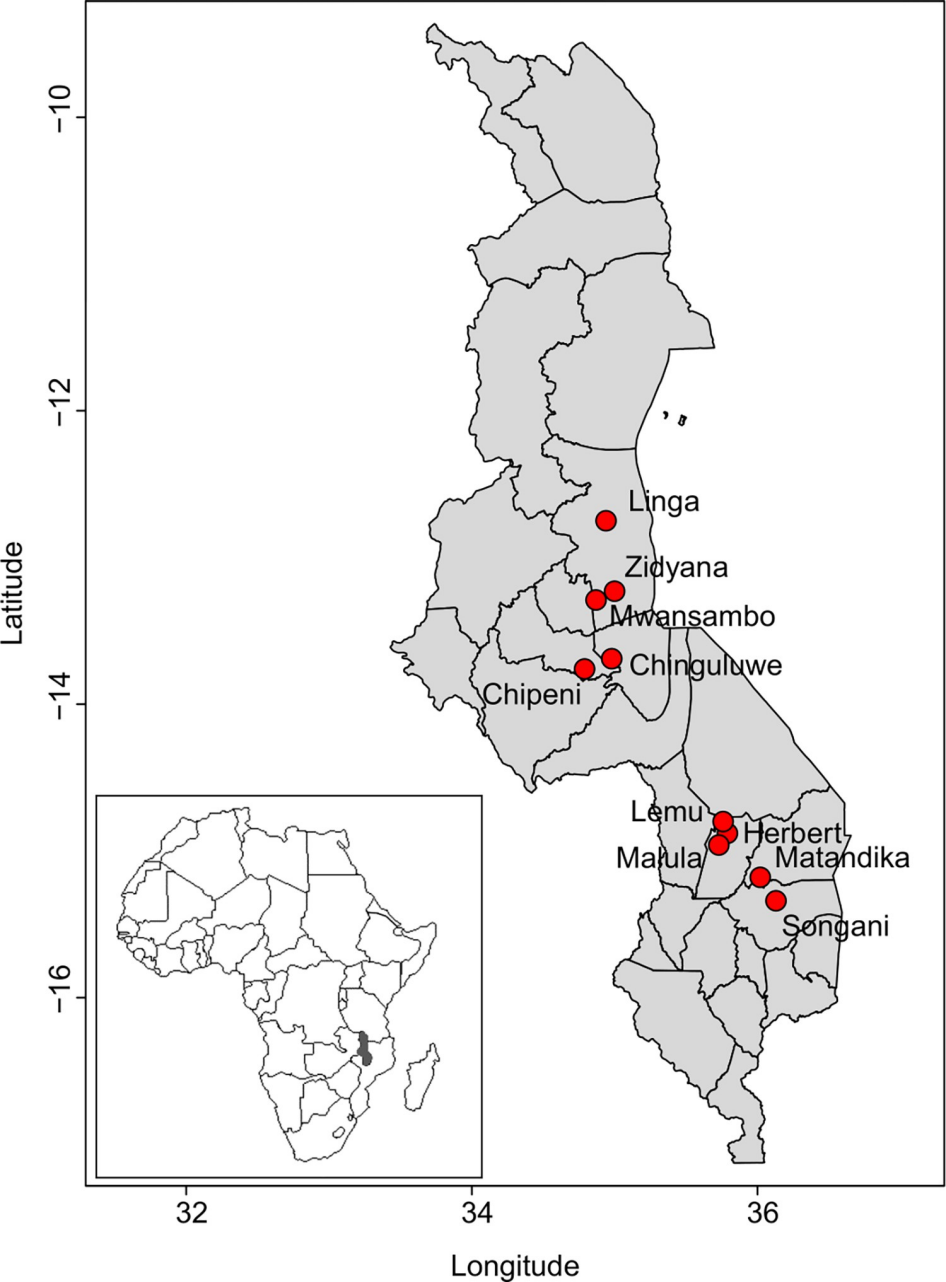
Geographical location of the ten communities in southern and central Malawi in which the study was conducted. The inset shows the position of Malawi in Africa.

The experiments were initiated in the 2005 growing season but for this current study, we present data from seven seasons only, i.e., 2013/2014 to 2019/2020 cropping seasons to avoid discrepancies in explaining varietal performance as maize varieties were maintained during this trial period. The study was carried out in on-farm experiments in ten communities located in six districts, Herbert, Malula and Lemu (in Balaka district), Chipeni (in Dowa district), Matandika (in Machinga district), Zidyana, Mwansambo and Linga (in Nkhotakota district), Chinguluwe (in Salima district), and Songani (in Zomba district) in central and southern Malawi. The geographical location, soil, and climate characteristics are given in Table 1 and Fig 1 and these have also been reported in previous research [24]. The soils in these communities are variable and categorized into two major soil groups: *Luvisols* (Malula, Lemu, Herbert, Matandika, Chipeni, and Zidyana) and *Lixisols* (Songani, Chinguluwe, Mwansambo, and Linga) [25].

**Table 1:** Province, district, geographical location, elevation, average seasonal temperature, and soil texture in the ten communities of Malawi.

Province	District	Community	Latitude (Decimal degrees)	Longitude (Decimal degrees)	Elevation (m asl) ¶	Average seasonal temperature (⁰C)	Soil texture description (0–30 cm)
Central	Dowa	Chipeni	-13.76	34.05	1166	21.5	Sandy loam
Central	Nkhotakota	Linga	-12.75	34.2	491	26.9	Sandy loam
Central	Nkhotakota	Mwansambo	-13.29	34.13	632	26.5	Sandy clay loam
Central	Nkhotakota	Zidyana	-13.23	34.26	535	26.9	Sandy clay loam
Central	Salima	Chinguluwe	-13.69	34.24	657	26.6	Sandy clay loam
Southern	Balaka	Herbert	-14.88	35.05	635	23.4	Sandy loam
Southern	Balaka	Lemu	-14.8	35.02	720	23.4	Sandy loam
Southern	Balaka	Malula	-14.96	34.99	605	23.4	Loamy sand
Southern	Machinga	Matandika	-15.18	35.28	688	23.4	Sandy loam
Southern	Zomba	Songani	-15.34	35.39	788	23.5	Clay loam

¶ m asl = metres above sea level

According to the Köppen climate classification, the communities Matandika, Songani and Chipeni experience dry winters and warm summers (Cwa) while the rest of the communities experience dry winters and hot summers (Cwb) [26].

**Fig 2:**
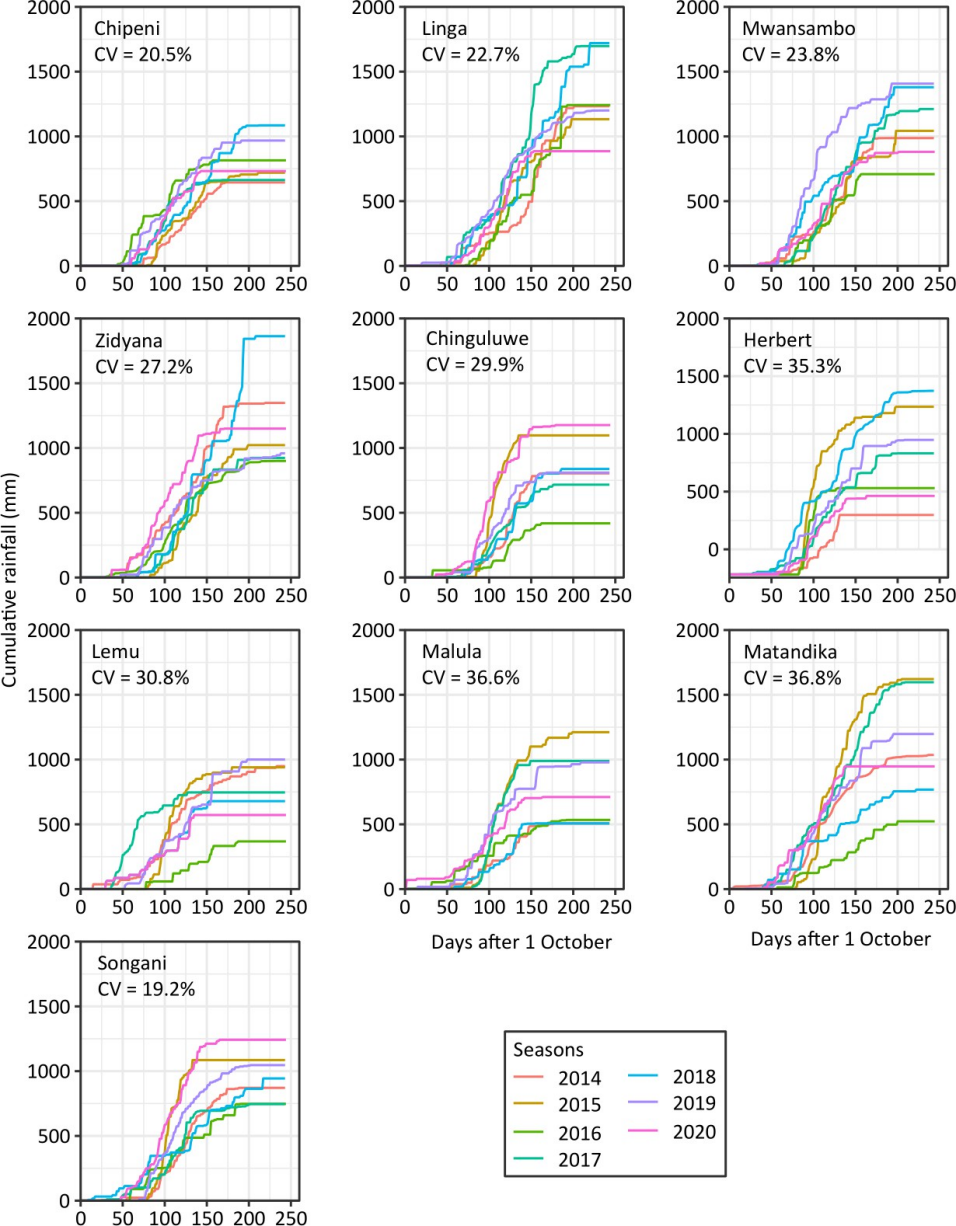
Cumulative seasonal rainfall received in the ten communities from 2013/2014 to 2019/2020. The coefficient of variation (CV) between the total seasonal rainfall are included for each site. Rainfall was recorded using rain gauges installed at each farmer site.

The area receives rainfall in a unimodal pattern and the annual average across all communities during the experimental period was 980 mm. This was received between November and April (Fig 2). Rainfall and temperature at the sites are described in detail in the results section 3.1. The area is characterized by mixed crop-livestock farming and smallholder farmers in this area predominantly grow maize (*Zea mays* L.) although crop diversifications with legumes such as groundnut (*Arachis hypogaea* L.), pigeonpea [*Cajanus cajan* (L.) Millsp] and to a certain extent soybeans (*Glycine max* L.) are also common.

### 2.2. Experimental set-up and management

At each trial community, the experiments were set at six farmers’ fields in a split-plot design with randomized blocks (RCB) using the one-farmer-one-replicate approach. This means that each farmers’ field was regarded as a replicate in the analysis. The farmers were chosen based on voluntary bases, but profiling was done for the representative farmers and for management purposes, six farmers were chosen for each community. At each farmers’ field (demonstration site hereafter referred to as “site”), three treatments (hereafter referred to as “systems”) were implemented, and these were:

- Conventional ridging (CPM-L)–here soil was tilled by manual labour and ridges formed which were rebuilt annually. Maize was sown as a sole crop and rotated annually with a legume. Crop residues were burned, incorporated into the soil, or removed from the field depending on customary practice by each individual farmer.
- No-tillage (CAM-L)–seeding into old ridges which were not reformed in subsequent seasons. Maize was sown as a sole crop and rotated annually with a legume. Crop residues were retained on the soil surface at the rate of approximately 2.5 t ha^–1^.
- No-tillage plus maize/legume intercropping (CAM/L-L)–seeding into old ridges which were not reformed in subsequent seasons. Maize intercropped with a legume but also rotated annually with a legume. Crop residues were retained on the soil surface at the rate of approximately 2.5 t ha^–1^.

For all systems, planting of maize was done by with a dibble stick (a pointed stick) on either annually formed ridges (for CPM) or previously formed ridges (for CAM-L and CAM/L-L). Although legumes are an important component of the cropping system, in this particular study we only considered the performance of maize. The legumes involved as rotational crops were groundnuts, pigeonpea, and cowpea (*Vigna unguiculata* Walp). In some cases, crop residues were burnt by mice hunters while the grazing policies that allow free roaming of cattle during the off-season resulted in the grazing of crop residues by roaming livestock especially in the southern region. In such cases, if all crop residues were burnt or grazed, crop residues for ground cover were imported at the rate specified for each treatment. In some cases, farmers would stack the residues out of the reach of livestock and then spread them at the beginning of the season.

The systems were established in plots measuring 1000 m^2^ and these were subdivided into 500 m^2^ and a rotation of maize and a legume was imposed in the subplots to have maize on one half and the legume on the other. Each maize plot was subdivided into five subplots and different maize varieties were sown in each subplot (Table 1). The varieties sown depended on the district and the exposure to drought and corresponding maturity length: However, in this current study we only focused on four genotypes of medium maturity length (Table 2), and these were:

- DKC 80–53 (Control variety)
- ZM 523
- MH 30
- PAN 53

**Table 2:** Source, maturity class, days to maturity, and vigour for the four genotypes tested across all environments for Balaka, Dowa, Machinga, Nkhotakota, Salima, and Zomba districts.

Genotype	Source	Maturity class	Days to maturity	Vigour ¶
DKC 80–53	Monsanto (K) Ltd	Medium	120	Hybrid
MH 30	Peacock Seeds	Medium	120	Hybrid
PAN 53	Pannar	Medium	120	Hybrid
ZM 523	Demeter	Medium	130	Improved OPV

¶ OPV = Open pollinated variety.

Maize was sown at the same rate in all districts and treatments with an interrow spacing of 75 cm and intrarow spacing of 25 cm to achieve a plant population of 53,333 plants ha^–1^ except for CAM/L-L from cropping season 2017/2018 onwards where treatments were seeded in 90cm × 25cm rows to achieve a target plant population of 44,444 plants ha^–1^. This was necessary to give room for the additional legume intercrop which could not proliferate in 75 cm rows. For further information on the rotational system and the different intercropping sequence please consult the exact treatment description in Komarek et al. [24].

All three treatments received a uniform fertilizer application rate of 69 kg N ha^–1^ which was applied in form of 100 kg of N:P:K ha^-1^ (23:21:0 + 4S) at planting and 100 kg urea ha^-1^ (46% N) at approximately three weeks after planting (total nutrient content applied was 69 kg N ha^–1^: 21 kg P_2_O_5_ ha^–1^: 0 kg K_2_O ha^-1^: 4 kg S ha^–1^) to the maize. No top-dressing was applied to the rotated and intercropped legumes. Weeds were controlled by spraying glyphosate [*N*-(phosphono-methyl) glycine], a pre-emergent herbicide, at the rate of 1.025 L active ingredient ha^–1^ immediately after sowing together with a residual herbicide Harness® [acetochlor (2-ethyl-6- methylphenyl-d11)] at a rate of 1 l ha^–1^. This was followed by manual hoe weeding whenever weeds were 10 cm tall or 10cm in diameter for stoloniferous weeds.

### 2.3. Yield data collection

For grain yield estimation, plants for each maize variety were harvested from each plot from an area of 15 m^2^ (two samples of 5 m × 1.5 m). Maize cobs were removed from the stalks and weighed for fresh weight, air-dried for about four weeks, and weighed again for dry weight. Grain moisture content was determined, and yield was expressed at 12.5% moisture content for maize. Maize stover were determined on a dry weight basis. Yield data were expressed per unit of surface area (ha).

### 2.4. Weather parameters

Rainfall was recorded using raingauges that were installed in farmers’ fields in all seasons at each community. Rainfall was recorded in the morning at around 8 am after a rainfall event and presented on a daily basis. Weather parameters that were not collected at each farmer field were obtained from the NASA Prediction of Worldwide Energy Resource (POWER) database (https://power.larc.nasa.gov/data-access-viewer/). The weather parameters obtained were (i) daily minimum and maximum air temperatures at 2m above the surface (⁰C), (ii) relative humidity (%), (iii) wind speed at 2m height above the soil surface (m s^–1^), (iv) all sky surface photosynthetic active radiation (W m^–2^), and (v) root zone soil wetness. Data from this site has been used in other studies carried out within the same communities as of our experiment [24]. We assume data from this NASA website is reasonably accurate and hence could be used in the study.

### 2.5. Calculations and statistical analysis

#### 2.5.1. Grain yield response to genotype, environments, management, and their interactions

To assess the genotype-by-environment (G×E) effects on maize grain yield under different management systems (M), we used linear mixed models using the ’lme4 package’ [27] in R environment [28]. In these models, G, M, and E (which was defined as the communities by season combinations) were considered as fixed variables while replicates (farmer fields), seasons, and cropping systems by replicates were considered as random variables. Significance of the fixed factors was tested using *F*-tests and where significances were detected, means were contrasted using mean comparison procedure based on Tukey test using ’emmeans’ package [29] in R.

To visualize the relationships between the genotypes and the environments; and management and environment, a genotype plus genotype by environment (GGE) biplot was used [30]. The GGE biplot was generated without transformation and scaling of data but were environment centred. Single value decomposition was then used to decompose the environment centred data into principal components (PC) [30, 31].

We constructed a which-won-where plot through joining the farthest genotypes creating an irregular polygon with each genotype on the vertex of the polygon. The performance (note that ‘performance’ in this study refers to yield) of the genotypes was determined by the position of the environments within the sectors such that a genotype performed the best in environments that were found in the same sectors [30]. The performance and the stability of the genotypes were also visualized using the average environment coordination (AEC) view of the GGE biplot for all the environments based on genotype-focused single value partitioning (SVP) [31]. Stability in this study has a dynamic meaning thus, when the conditions within a particular environment were conducive, yield was higher and vice versa. This implies that genotypes that were highly stable had yield higher than other genotypes in relation to the average yield of all genotypes across all the environments. All analyses were done using the ’metan’ package in R.

#### 2.5.2. Yield response to seasonal weather parameters

To assess the response of the genotypes and cropping systems (management) to weather parameters, we used the weather parameters as the explanatory variables. Seasonal rainfall was calculated by summation of all daily rainfall during the growing season for each season (i.e., from 1^st^ October to 31^st^ May) and seasonal temperature was calculated by first calculating the daily average temperature based on the maximum and minimum temperature and then averaging the daily temperatures for each growing season in each community. For all the other weather parameters, daily readings were averaged across the season and used in the analyses. We employed regression analysis to test the effect of the weather parameters on maize grain yield. First, we ran several models with only first-order polynomials, second-order polynomials, or both for each continuous variable based on previous studies and then employed model selection using the Akaike Information Criterion (AIC). The purpose of this step was not to describe the curve of response of the different variables but to show a curve relationship better explained the response than a linear response. The AIC test showed that second-order polynomials better explained the response than linear responses for all parameters in the models. Since rainfall and air temperature are the most critical weather parameters in the region [32], we focused more on dissecting their effect on maize grain yield for each cropping systems and each genotype. Based on previous work, e.g., Komarek et al. [24], Rusinamhodzi et al. [33], and Hoffman et al. [34] which highlighted that the relationship of grain yield with rainfall and air temperature may follow an inverted U-shaped relationship, we fitted an orthogonal quadratic regression with a second-degree polynomial. Models were estimated separately for each genotype and for each management system, i.e., one regression model was run for each genotype and for each management system by taking a subset of the data from the entire dataset.

#### 2.5.3. Hybrid and Conservation Agriculture system yield response

To assess the response of hybrids in comparison to the OPV, and that of CA-based systems to that of CPM-L, we calculated best linear unbiased predictors (BLUPs) of the ‘genotype × environment’ and ‘management × environment’ interactions, respectively. The BLUPs were extracted from the linear models that were fit which had either management or genotype as the fixed effects while season, replicates (farmer fields), and the interaction of either management or genotype with environment as random effects. BLUPs are more centred towards the overall mean across the environments hence more desirable than the actual means [35]. Linear models were used to express the BLUPs of the hybrids and CA-based systems as a function of the OPV and the CPM-L system, respectively. The intercepts and slopes (estimates) were extracted from the linear models, and these were used to construct the regression line against the 1:1 line. A regression line above the 1:1 would signify higher yields of hybrids or CA-based systems and vice versa.

## 3. Results

### 3.1. Seasonal weather parameters during the season in the communities

The communities received variable rainfall during the study, and it ranged from 368 mm in the worst case (which was received in Lemu) to 1863 mm (which was received in Zidyana community) (Fig 2). Across the seasons, rainfall was quite variable in all communities with coefficient of variation ranging between 19.2% and 36.8% (Fig 2 and S1 Fig). Rainfall was generally highest in the Linga community across the seasons and the mean rainfall received was 1380 mm (Fig 2). Mid-season droughts varied in length across the sites and across the seasons within a site and these ranged between 10 days to an excess of 30 days. For example, in Herbert, the season 2015 had very short mid-season dry spells while in other seasons were very long (20 days or more) (Fig 2). Overall, the level of variability in rainfall depended on the community. Due to the resolution of the website, sites that were close to each other had same values for the other weather parameters. Thus, for temperature which we used for further analyses only temperatures of representative sites were presented (S2 Fig). The lowest average daily minimum temperature across all sites was 19.6 ⁰C while the highest was daily maximum temperature was 39.6 ⁰C. Average seasonal temperature ranged being 21.5 ⁰C and 26.9 ⁰C across the sites. An attempt to cluster the sites into three elevation levels, i.e., low (<500 m asl), middle (500>800 m asl), and high (>800 m asl) altitude resulted in similar temperatures within the levels and across the levels. Relative humidity (RH) average 68.3% across the sites with the lowest being 63.9% observed at Zidyana and the highest was 74.1% at Chipeni and both sites were located in Central Malawi (S1 Table). For all sky surface photosynthetic active radiation (PAR) the variation across the sites was not huge (CV = 1.7%) and ranged between 110.7 W m^–2^ and 114.5 W m^–2^ across the sites. Wind speed considerably varied across the sites (CV = 27.2%) with the highest wind speed being observed in Central Malawi in Linga which recorded 4.1 m s^–1^. Root zone soil wetness (RZW) was relatively similar across all the sites (CV = 6.6%) and ranged between 0.7% and 0.8% (S1 Table).

### 3.2. Genotype, environment, and management and their interaction effects on grain yield

There were significant differences in all fixed factors and their interactions (e.g., G×E and E×M), except for the G×M and G×E×M interactions (Table 3). Most of the variation on grain yield was explained by the management systems followed by the environments (Table 3). None of the interactive effects accounted for more variation than the main effects of G, E, and M.

**Table 3:** Effects of genotypes, environments, and management on maize grain yield for four genotypes tested across all the environments.

Source	Sum of squares	Degree of freedom	*F*-value
Genotype (G)	1.1 × 10^8^	3	21.0***
Environment (E)	5.4 × 10^9^	68	43.7*
Management (M)	3.9 × 10^8^	2	108.9***
G × E	4.4 × 10^8^	204	1.2*
G × M	1.3 × 10^7^	6	1.2^NS^
E × M	8.1 × 10^8^	136	3.3***
G × E × M	3.2 × 10^8^	408	0.4^NS^

F-values followed by asterisks show significant differences at

* and *** representing *P* < 0.001 and *P* < 0.05, and

^NS^ means not significant.

**Fig 3:**
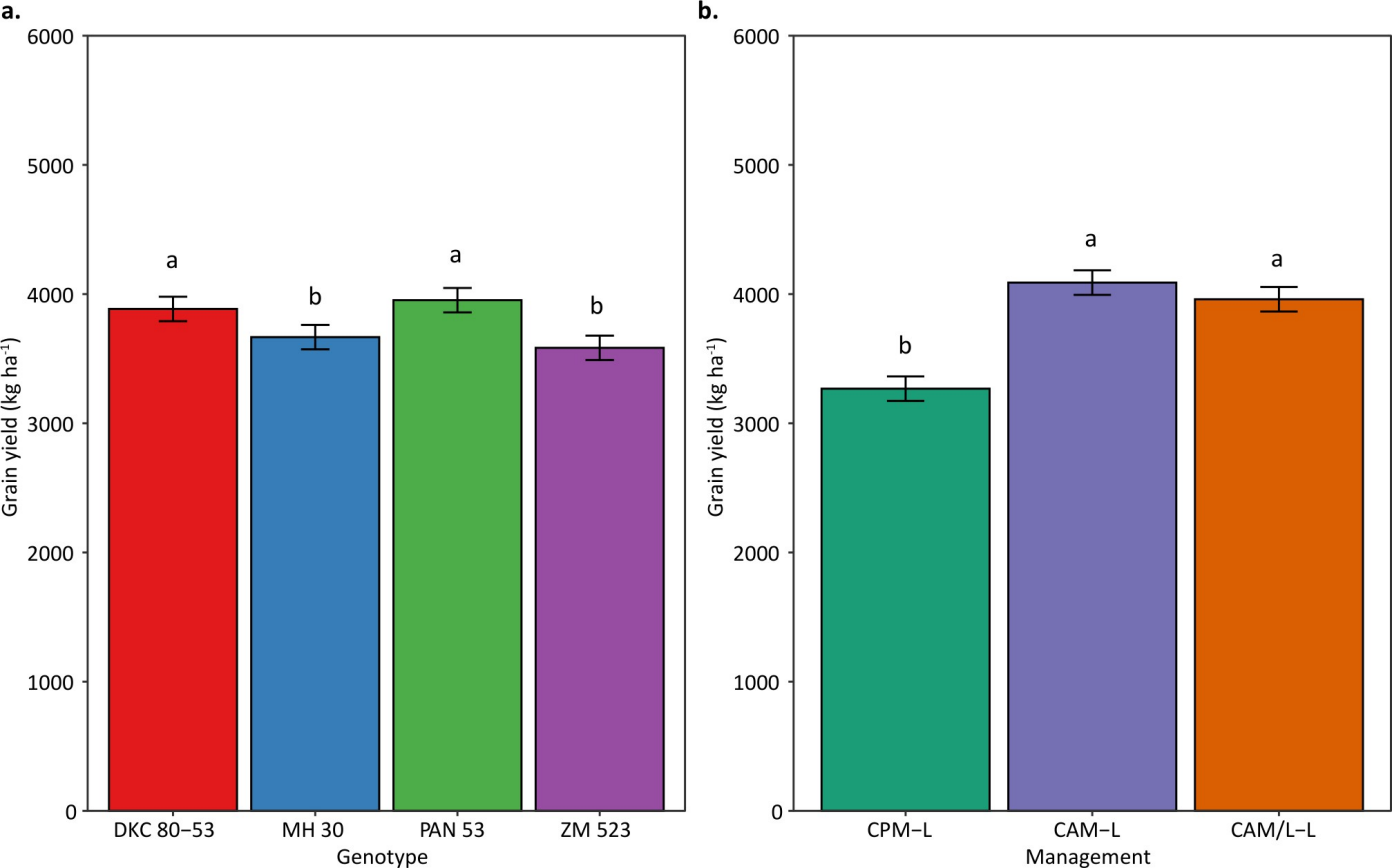
Genotype and management yield variation across the environments. Column bars with different letters above them are significantly different means at 5% probability level. The error bars represent the standard error of the mean. Management systems abbreviation: CPM-L = Conventional ridging and maize monocropping; CAM-L = Reduced tillage and maize monocropping; and CAM/L-L = Reduced tillage plus intercropping.

The control genotype DKC 80–53 and PAN 53 yielded the highest grain averaging 3850 kg ha^–1^ while the OPV ZM 523 together with the hybrid MH 30 yielded the least (Fig 3A). Conservation agriculture without intercrop (CAM-L) as well as with intercropping (CAM/L-L) yielded the highest (with an average yield of 3800 kg ha^–1^) while the conventional system had the lowest (3200 kg ha^–1^) yield (Fig 3B).

**Fig 4:**
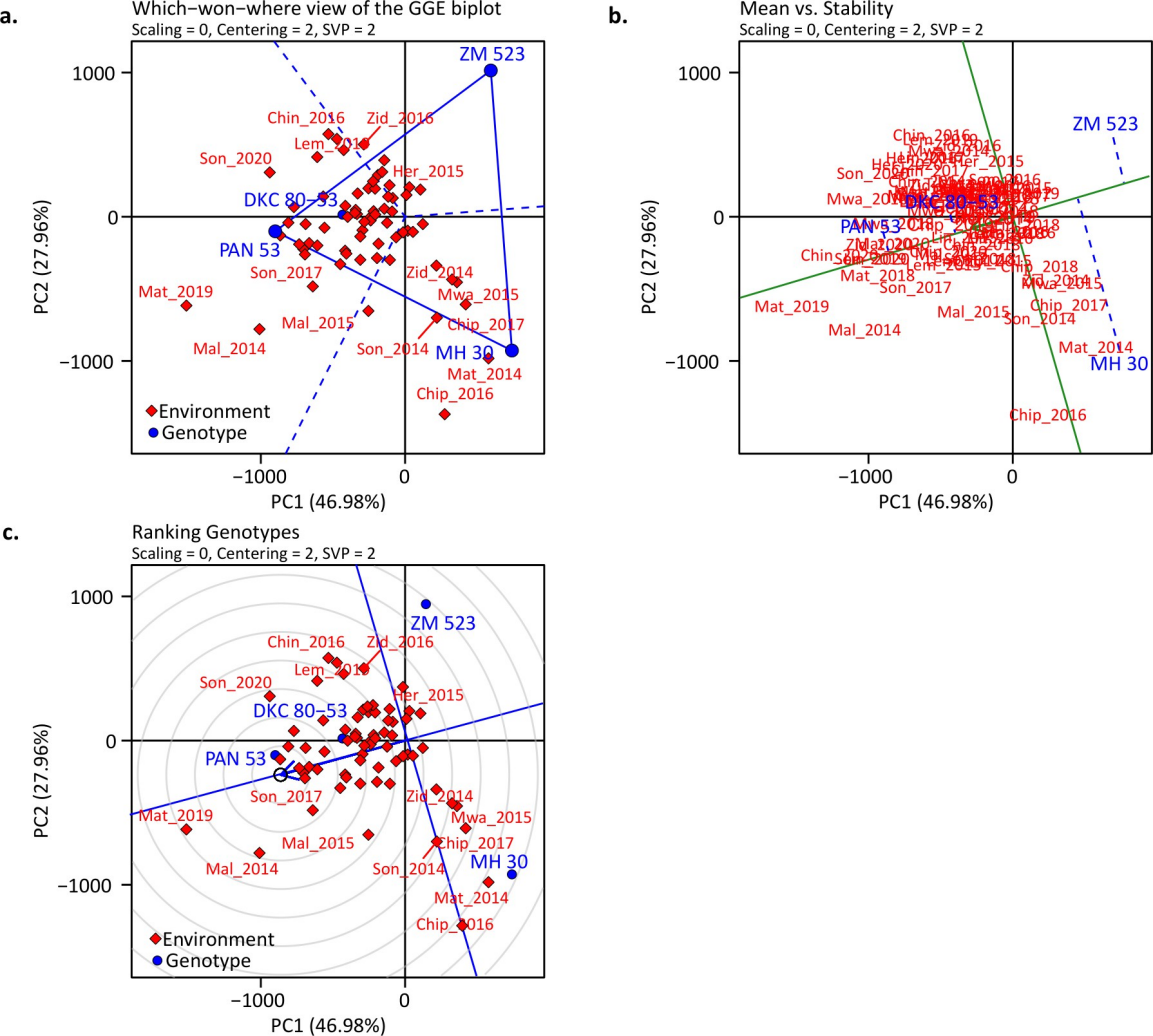
The which-won-where view of the genotype plus genotype by environment (a), average environment coordination (mean versus stability) (b), and genotype ranking (c) for the five genotypes tested in all environments in the Balaka, Dowa, Machinga, Nkhotakota, Salima, and Zomba districts. The data were not scaled (“*Scaling* = 0”) and environment centred (“*Centering* = 2”), and with environment-focused single value partitioning (“*SVP* = 2”) for all plots. The environments were based on the combination of communities and years, i.e., the first three letters of the community’s name and the season where: Her = Herbert; Mal = Malula; and Lem = Lemu; Chin = Chinguluwe; Chip = Chipeni; and Lin = Linga; Mat = Matandika; Mwa = Mwansambo; Son = Songani; and Zid = Zidyana.

The GGE biplot explained 74.9% of the grain yield variation observed for the G and G × E (Fig 4). The which-won-where biplot (Fig 4A) revealed that only three (PAN 53, ZM 523, and MH 30) of the four tested genotypes were on the vertex, indicating their superior or inferior performance in some or all the environments that were within their sectors. The hybrids PAN 53 and DKC 80–53 were the best performers in most of the environments they were tested in, while the OPV ZM 523 only performed well in a few environments characterized by above-average rainfall and shorter mid-season droughts compared to others e.g., Herbert 2015 (Fig 4A). The hybrid MH 30 also exhibited considerable performance in many environments. On top of their superior performance in majority of the environments, a comparison of stability showed the hybrids PAN 53 and DKC 80–53 to be the most stable while OPV ZM 523 and hybrid MH 30 were least stable (Fig 4B).

**Fig 5:**
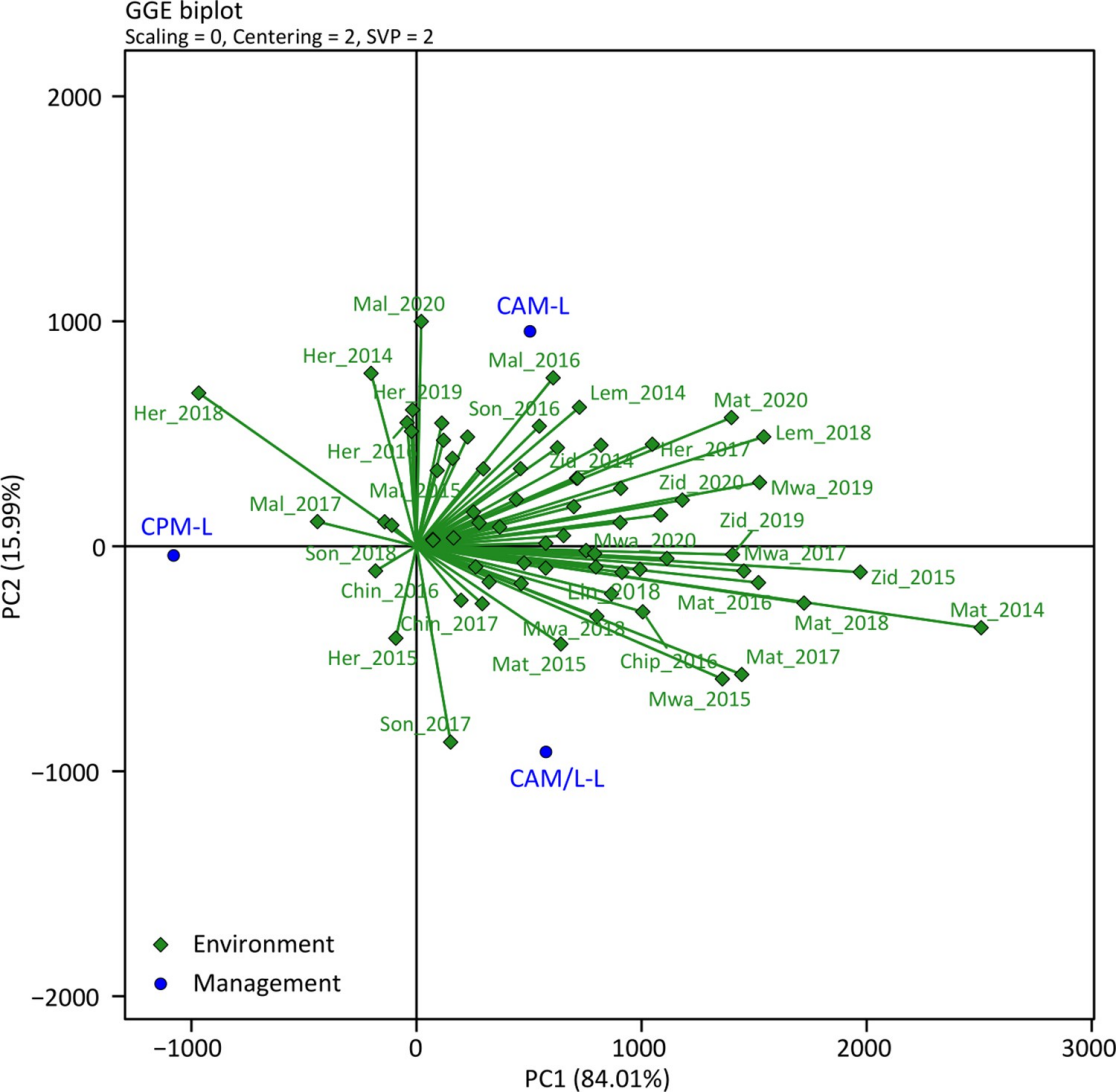
Biplot view of the relationship between management and environments for the three management systems tested in all environments in the Balaka, Dowa, Machinga, Nkhotakota, Salima, and Zomba districts. The data were not scaled (“Scaling = 0”) and environment centred (“Centering = 2”), and with environment-focused single value partitioning (“SVP = 2”) for all plots. The environments were based on the combination of communities and years, i.e., the first three letters of the community’s name and the season where: Her = Herbert; Mal = Malula; Lem = Lemu; Chin = Chinguluwe; Chip = Chipeni, and Lin = Linga; Mat = Matandika; Mwa = Mwansambo; Son = Songani; and Zid = Zidyana. Management systems abbreviation: CPM-L = Conventional ridging and maize monocropping; CAM-L = Reduced tillage and maize monocropping; and CAM/L-L = Reduced tillage plus intercropping.

A ranking of the genotypes based on mean performance and stability put the genotypes in the following order: PAN 53 > DKC 80–53 > ZM 523 > MH 30 showing that the hybrid PAN 53 was superior in terms of performance and stability due to its position on the concentric rings while the hybrid MH 30 was the most inferior (Fig 4C). As for the management systems, the CAM-L and CAM/L-L systems were associated with most of the environments as compared to the CP system (Fig 5). The CPM-L system performed well mainly in the high rainfall environments, e.g., Herbert in 2018 and Malula in 2017 (Fig 5). These environments were also characterized by short mid-season dry spells of about 15 days or less which also occurred outside of critical growth stages (Fig 2).

### 3.3. Relationship of grain yield with weather parameters for the different genotypes and management systems

All investigated weather parameters had a significant effect on maize grain yield across all the environments for both the linear and quadratic terms except for PAR which was insignificant for both as shown in S2 Table. Maize yield response was variable across the parameters with a concave response being noted for all parameters except for air temperature and RZW which showed a convex response (S3 Fig and S2 Table).

An investigation of genotype and management system response to rainfall and temperature showed that the genotypes and management systems responded differently to seasonal rainfall across the environments (Table 4).

**Table 4:** The effects of seasonal rainfall on maize grain yield for each investigated genotype and management system.

Genotype/ Management	Source	Estimate	Standard error	t value	Pr(>|t|)
DKC 80–53	(Intercept)	4087.7	60.27	67.819	< 2e-16 ***
	poly(Rainfall, 2)1	2316.71	1780.89	1.301	0.194
	poly(Rainfall, 2)2	-10089.28	1780.89	-5.665	1.99e-08 ***
MH 30	(Intercept)	3889.4	59.14	65.771	< 2e-16 ***
	poly(Rainfall, 2)1	926.21	1747.26	0.53	0.596
	poly(Rainfall, 2)2	-8120.91	1747.26	-4.648	3.87e-06 ***
PAN 53	(Intercept)	4147.55	60.13	68.973	< 2e-16 ***
	poly(Rainfall, 2)1	-473.13	1777.73	-0.266	0.79
	poly(Rainfall, 2)2	-7995.16	1777.73	-4.497	7.81e-06 ***
ZM 523	(Intercept)	3774.66	56.15	67.222	< 2e-16 ***
	poly(Rainfall, 2)1	1667.04	1658.16	1.005	0.31501
	poly(Rainfall, 2)2	-4649.74	1658.16	-2.804	0.00516 **
CPM-L	(Intercept)	3365.39	47.19	71.319	< 2e-16 ***
	poly(Rainfall, 2)1	-2400.34	1614.07	-1.487	0.137
	poly(Rainfall, 2)2	-8984	1614.07	-5.566	3.23e-08 ***
CAM-L	(Intercept)	4262.76	51.14	83.357	< 2e-16 ***
	poly(Rainfall, 2)1	2954.8	1739.47	1.699	0.0897 .
	poly(Rainfall, 2)2	-10391.81	1739.47	-5.974	3.08e-09 ***
CAM/L-L	(Intercept)	4301.25	50.43	85.298	< 2e-16 ***
	poly(Rainfall, 2)1	3282.72	1721.15	1.907	0.0567 .
	poly(Rainfall, 2)2	-7445.7	1721.15	-4.326	1.65e-05 ***

*P*-values followed by asterisks show significant differences at

* and *** representing P < 0.001 and P < 0.05, and

NS means not significant.

**Fig 6:**
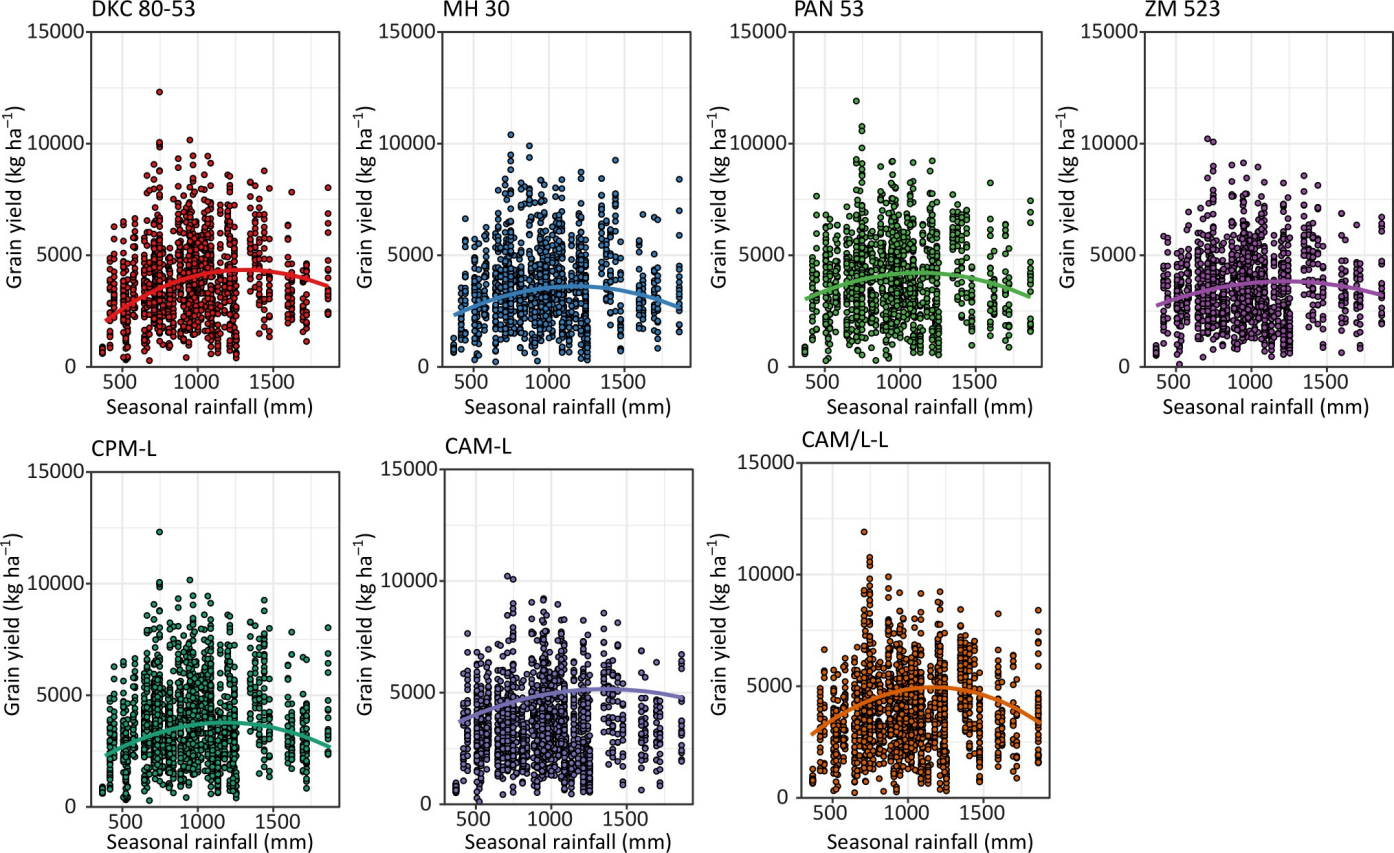
Responses of genotypes and management systems to seasonal rainfall measured from 2014 to 2021 across all the environments for Balaka Dowa, Machinga, Nkhotakota, Salima, and Zomba districts. Lines were constructed using second degree orthogonal regression using retained models. The scatter points represent the raw data. Management systems abbreviation: CPM-L = Conventional ridging and maize monocropping; CAM-L = Reduced tillage and maize monocropping; and CAM/L-L = Reduced tillage plus intercropping.

For all genotypes and management systems, seasonal rainfall resulted in a concave response on grain yield with yields reaching a plateau and declining as seasonal rainfall increased over a certain level (Fig 6). The genotypes PAN 53 and DKC 80–53 showed the highest crests, and this also supports their high yields across the environments, while the genotypes ZM 523 and MH 30 showed lower crests. The more flattish curve on ZM 523 could signify its poor stability performance across the environments. As for management system, the CPM-L system showed the lowest peak response as compared to the CAM-L and CAM/L-L systems (Fig 6). The CAM/L-L system showed the highest peak of response signifying its high response to seasonal rainfall.

**Fig 7:**
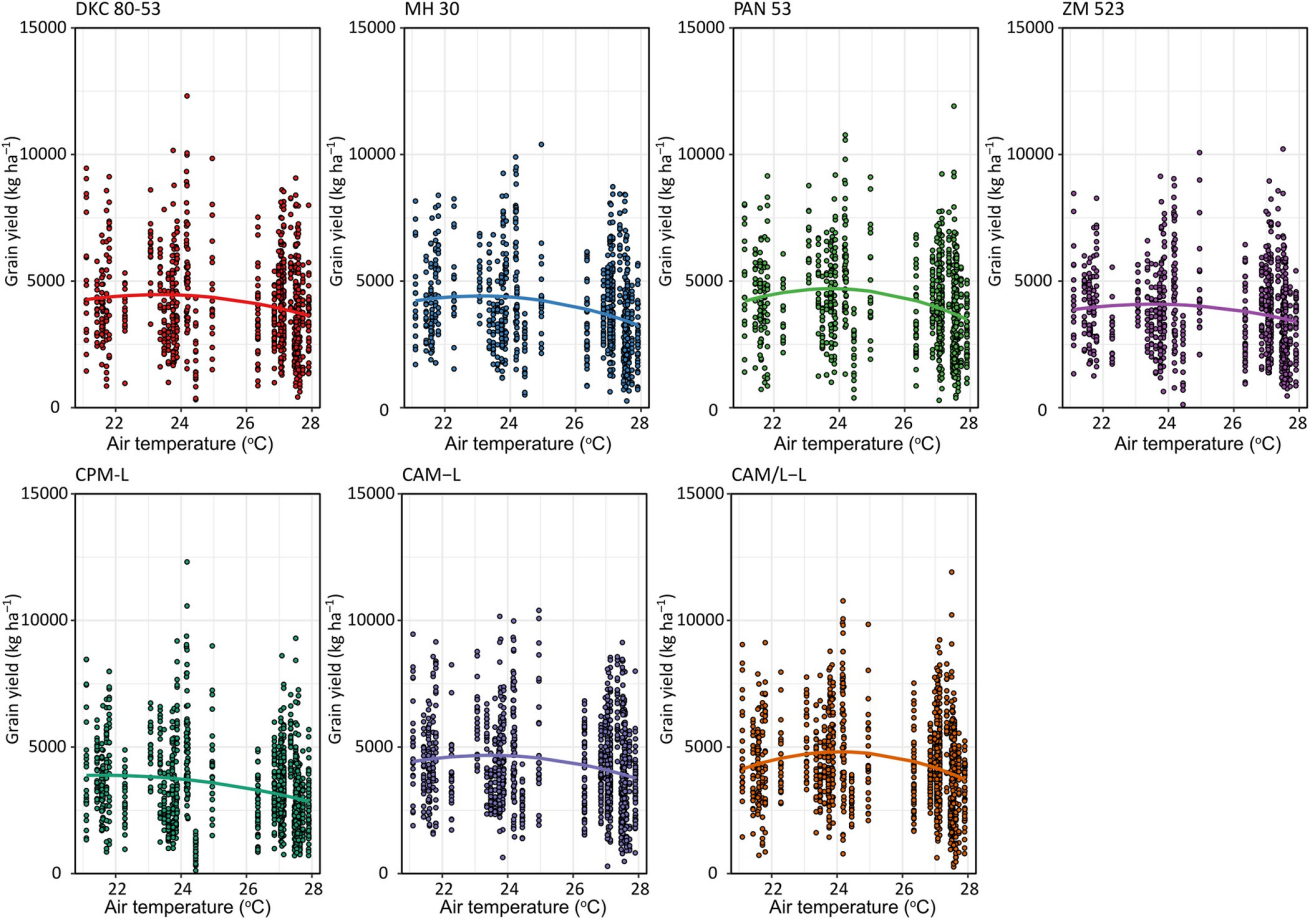
Responses of genotypes and management systems to seasonal air temperature measured from 2014 to 2021 across all the environments for Balaka Dowa, Machinga, Nkhotakota, Salima, and Zomba districts. Lines were constructed using second degree orthogonal regression using retained models. The scatter points represent the raw data. Management systems abbreviation: CPM-L = Conventional ridging and maize monocropping; CAM-L = Reduced tillage and maize monocropping; and CAM/L-L = Reduced tillage plus intercropping. Temperature data was acquired from the NASA Power website https://power.larc.nasa.gov/data-access-viewer/.

Average air temperature had a significant effect on the response of all the genotypes for both the linear and the quadratic terms (Table 5). The response of the different genotypes was variable with PAN53 having the highest peak of response as comapred to the other genotypes (Fig 7). The OPV ZM 523 also showed the lowest peak of response as was observed for the rainfall response. As for management systems, the CAM/L-L system responded the most to air temperature followed by the CAM-L systems and lastly the CPM-L system which showed a visibly flattish and declining curve (Fig 7).

**Table 5:** The effects of seasonal air temperature on maize grain yield for each investigated genotype and management system.

Genotype/ Management	Source	Estimate	Standard error	t value	Pr(>|t|)
DKC 80–53	(Intercept)	4087.7	60.59	67.462	< 2e-16***
	poly(Air temperature, 2)1	-7857.47	1790.31	-4.389	1.28e-05***
	poly(Air temperature, 2)2	-4018.26	1790.31	-2.244	0.0251*
MH 30	(Intercept)	3889.4	58.17	66.863	< 2e-16***
	poly(Air temperature, 2)1	-11323.17	1718.73	-6.588	7.71e-11***
	poly(Air temperature, 2)2	-4963.33	1718.73	-2.888	0.00398**
PAN 53	(Intercept)	4147.55	59.19	70.072	< 2e-16***
	poly(Air temperature, 2)1	-9966.6	1749.85	-5.696	1.68e-08***
	poly(Air temperature, 2)2	-7103.8	1749.85	-4.06	5.36e-05***
ZM 523	(Intercept)	3774.66	55.83	67.605	< 2e-16***
	poly(Air temperature, 2)1	-6172.2	1648.75	-3.744	0.000193***
	poly(Air temperature, 2)2	-3655.37	1648.75	-2.217	0.026877*
CPM-L	(Intercept)	3365.39	46.55	72.301	< 2e-16***
	poly(Air temperature, 2)1	-12636.61	1592.14	-7.937	4.84e-15***
	poly(Air temperature, 2)2	-2972.38	1592.14	-1.867	0.0622 .
CAM-L	(Intercept)	4262.76	51.13	83.377	< 2e-16***
	poly(Air temperature, 2)1	-9540.39	1739.04	-5.486	5.05e-08***
	poly(Air temperature, 2)2	-5237.82	1739.04	-3.012	0.00265**
CAM/L-L	(Intercept)	4301.25	49.83	86.311	< 2e-16***
	poly(Air temperature, 2)1	-8327.58	1700.94	-4.896	1.12e-06***
	poly(Air temperature, 2)2	-8786.38	1700.94	-5.166	2.82e-07***

*P*-values followed by asterisks show significant differences at

* and *** representing P < 0.001 and P < 0.05, and

NS means not significant. Temperature data was acquired from the NASA Power website https://power.larc.nasa.gov/data-access-viewer/.

### 3.4. Hybrid and Conservation Agriculture system yield response

**Fig 8:**
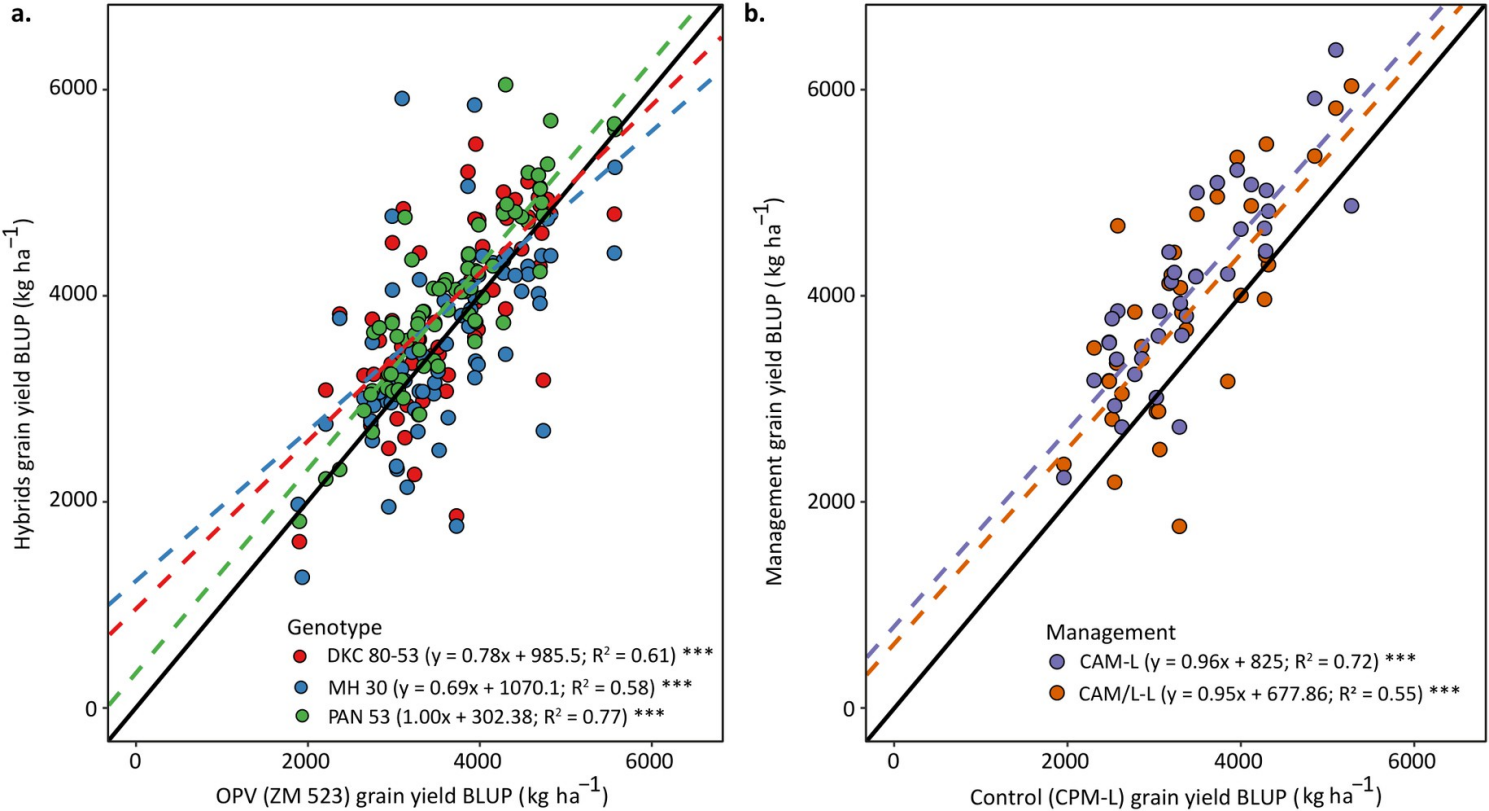
Best linear unbiased predictors (BLUPs) of yield of the hybrids DKC 80–53, MH 30, and PAN 53 compared with the open-pollinated variety ZM 523 (a); and BLUPs of the conservation agriculture-based management practices CAM-L and CAM/L-L compared with the control practice CPM-L (b). The diagonal solid black lines represent the 1:1 relationship. The dashed lines represent the fitted regression using linear modeling and each scatter point represents the BLUPs for each observation for the interaction of the genotype or the management systems with the environment. Management systems abbreviation: CPM-L = Conventional ridging and maize monocropping; CAM-L = Reduced tillage and maize monocropping; and CAM/L-L = Reduced tillage plus intercropping.

There was a significant and positive relationship between the yields of the different hybrids and the OPV although the relationship was not very strong (Fig 8A). For the PAN 53 hybrid, the yield response was positive across the environments as evidenced by most of the data points above the 1:1 line as well as by the regression line that was consistently above the 1:1 line throughout the yield levels (Fig 8A). However, for the DKC 80–53 and the MH 30 hybrids, there was a ‘cross-over’ effect of hybrid performance as compared to the OPV. At lower yield levels, yield response of the hybrids was higher than that of the OPV, but the performance became negative as yield levels were above 4000 kg ha^–1^ (Fig 8A). As for the management, the implementation of CA-based systems (CAM-L and CAM/L-L) resulted in positive yield responses across the environments (Fig 8B). However, the CAM-L system had a higher yield response compared to the CAM/L-L system.

## 4. Discussion

### 4.1. Genotype, environment and management and their interaction effects on grain yield

The performance of agriculture crops depends on many factors, and these include the environmental conditions, the management, the type and genotypic potential of the crops amongst others. The environmental conditions aspect consists of many facets such as soil characteristics and weather conditions etc. Although weather parameters such as rainfall, air temperature, relative humidity, wind speed, and root zone soil wetness amongst others are important, here we dwelled on the parameters which are most important in southern Africa i.e., rainfall and temperature [32]. Moreover, other previous studies have already used indices that combine all the above-mentioned parameters (please see Komarek et al. [24]) and hence our focus on this current study was on the two most important in the region which are rainfall and temperature. Despite having the knowledge of how crops respond to the aforementioned aspects (i.e., environment, management, and genetics) separately, understanding how the interaction of these aspects affects maize yield is important. Here, we assessed the performance of four genotypes (three hybrids and one OPV) managed under three different CA-based practices across ten on-farm communities of central and southern Malawi. Our results showed that across the environments and management systems, hybrids had higher yields as compared to the OPV of the same maturity group (Fig 3A). Such hybrids that performed well, even better than other hybrids, were the drought-tolerant PAN 53, and the control hybrid DKC 80–53 (Fig 3A). From previous research, we already know that hybrids will likely yield more than OPVs under favourable conditions [36] but this depends on whether the hybrids are well adapted to the environmental conditions and of course the management practices applied (e.g., fertilization level, tillage, residue retention and diversification strategy etc). The higher yields normally observed in hybrids are mainly due to their genes being expressed in a heterozygous manner, thus there is an accumulation of dominant favourable genes [36–38]. As shown by more favourable responses to seasonal precipitation and air temperature (Figs 6 and 7), hybrids have higher resource accumulation and utilization and hence show greater productivity as compared to OPVs although this was only evident until a specific rainfall and temperature threshold and afterwards it declined. Such curves of response of maize to rainfall have also been reported by Rusinamhodzi et al. [33]. Hybrids have shown more tolerance to heat stress as compared to the OPV with higher thresholds (Fig 7). These responses may be attributed to a combination of improved root architecture, improved resource partitioning, and improved adaptation to abiotic and biotic stresses [39]. This often results in more uniform grain in terms of size and hence higher yield. Improvement in maize grain yield of up to 35% due to the adoption of hybrids has been reported in southern Africa in previous studies [11, 39, 40]. Contrary to our findings, Tulu and Jifar [41] showed that OPVs yielded higher than hybrids under six of the environments in an above-average rainfall area signifying their superiority in yield-limiting or unfavourable or too wet environments. Also, important to note is the importance of rainfall distribution on top of the total received. Mid-season dry spells of only 10 days during critical growth stages such as flowering and grain filling can lead to 70% yield losses [42] meaning that understanding rainfall distribution can help explain the differences observed by the two studies.

The existence of a genotype by environment interaction signifies that the performance of genotypes depends on the environmental conditions (Table 3). This indicates that optimizing genotypes for certain environments is pivotal for improving yield although this can complicate genotype selection for broad adaptation [43]. A study by Seyoum et al. [44] that unveiled the physiological processes underpinning G×E×M interaction effects on maize yield showed that environments have a huge influence in yield determination as compared to genotypes and management.

The GGE biplots in our study showed that hybrids performed well in most of the environments, and this may be attributed to their hybrid vigour (heterosis) attained from improved genetics which makes them adapted to most environments in the study (Fig 4). Based on our findings, we can accept the first hypothesis that hybrids yield more than OPVs across different environments and reject the second hypothesis that a G×E×M interaction determines yield in this context since we only observed a G×E and E×M effect. This means that farmers in the communities of Malawi should choose maize varieties that are suited to their environmental conditions to enhance maize productivity under the smallholder farming systems in different environments. However, this finding does not completely dismiss the presence of a genotype by environment by management interactive effect in yield determination, but only illustrates its absence in the context within which the study was conducted. Despite previous reports that OPVs are generally more stable than hybrids, the OPV in our study showed less yield stability across the environments as compared to the hybrids PAN 53 and DKC 80–53 (Fig 4B). The OPV in the study was generated by crossing some of the elite maize lines during hybrid breeding and then tested across a fewer optimal and stress environments [45]. This normally results in a less intense selection process and hence a higher genetic variability as compared to a hybrid selection pipeline. Thus, due to the more intense selection in the hybrid breeding pipeline, the hybrids in the study were more adapted to most of the Malawian environments and hence more stable. On the other hand, the more flattish curve of response to seasonal precipitation and temperature (Figs 6 and 7) exhibited by the OPV indicated that its yield was almost similar across the environments, thus less responsive to the more optimal environmental indices. This could be defined as static stability which means that in the face of the ever-changing climate, such a genotype will guarantee a statically stable yield although not utilizing the more conducive environments. However, OPVs are generally more tolerant to common diseases such as grey leaf spot (*Cercospora zeae-maydis*) and common rust (*Puccinia sorghi*) which are prevalent in the study area and such qualities ensure reasonable yields across different environments. Despite the lower yield and lower stability from the OPV, it is a multiple line synthetic that can be recycled for 3–4 cropping seasons with lower yield penalties as compared to recycling hybrids and hence an attractive option for resource-constrained smallholder farmers in southern Africa [45, 46]. However, this depends on the degree of isolation from contamination by pollen from other varieties, a practice that could be difficult for smallholder farmers. We therefore partially reject the hypothesis that OPVs are more stable than hybrids since this depends on the hybrid to which they are being compared.

As for cropping system performance (management) across all genotypes and environments, the direct-seeded treatment plus maize monocropping (CAM-L) either outperformed all management systems or was at par with the direct-seeded treatment with intercropping (CAM/L-L) while the conventional ridging (CPM-L) was low yielding in most of the environments (Figs 3B and 5). This may be attributed to the continuous ridging in all seasons in the CPM-L system slowly degrading soil quality. Continuous tillage has been shown in many instances to pulverise the soil and hence resulting in poor soil aggregate structure leading to poor infiltration and other water related properties [47, 48]. This was reflected in the poor responses of the CPM-L system to seasonal precipitation and temperature (Figs 6 and 7), which could mean that water in this system was not captured and utilized by the crops but rather was lost as run-off and also the crops in this system did not respond to temperature for relevant processes. Thus, this system was not effective in curbing the devastating effects of mid-season dry spells and heat stress by not conserving water which could be used by plants during these periods. This is very important since rainfall and temperature are generally erratic in southern Africa. Continuous tillage can also disrupt the micro- and macrofaunal communities in the soil and this can lead to compromised ecosystem services from these communities [49–51]. For example, a study by Bowles et al. [52] showed that tillage can disrupt mycelial networks of arbuscular mycorrhizal fungi (AMF), and this results in reduced colonization of crop roots and hence reduced nutrient acquisition. A recent study by Mhlanga et al. [22] showed that mulching is important in cropping systems of southern Africa as it serves as a source of organic matter for increased microbial activity as well as conserving moisture. This was also confirmed by Thierfelder et al. [53]. Since the CPM-L system had no mulch, organic matter build-up, moisture conservation and soil temperature regulation in this system were not enhanced as compared to the CA-based systems. Although we expected the CAM/L-L to yield more than the CAM-L system over time due to the additional intercropped legume, the yield was either lower or equivalent (Fig 3B). This may be due to the competition between the maize and the legumes which outweighed the benefits of the intercrop. In addition, the lower plant population on this treatment as required to accommodate the intercropped pigeonpea or cowpea had an impact on the final yield as maize crops could not compensate yield in the lower population density. Our results are in accordance with previous findings from Madembo et al. [54] in a study from Zimbabwe which clearly highlighted that intercrops usually led to yield penalties on maize once intercropped. Such negative competition may be alleviated by supplementing resources such as fertilizer or by optimizing planting patterns to reduce the competition. However, despite the reduced maize yields due to competition in intercrops, total system yield has been shown to be increased when both crops in the intercropped system are being combined [55, 56] leading to a higher land equivalent ratio [57]. In summary and based on our findings, we can accept the first, third, and fifth hypotheses that hybrids and CA-based systems yield more and are more responsive to seasonal rainfall and temperature than OPVs and CP-based systems, respectively.

### 4.2. Hybrid and Conservation Agriculture system yield response

Of the hybrids tested in our study, PAN 53 showed consistently higher yields as compared to the OPV at all yield levels (Fig 8A). As for the other hybrids, DKC 80–53 and MH 30, the yield was higher at low yield levels but then declined at higher yield levels. The hybrid PAN 53 is a robust and drought tolerant hybrid which is more adapted to the prevailing conditions of Malawi. This allows it to yield considerably well across many environments. PAN 53 has been reported to have a high seed vigour which results in higher germination and rapid seedling establishment leading to uniformity and robustness across diverse environmental conditions [56–59]. PAN 53 is also the most widely grown hybrid in Zambia promoted there under the slogan “Shanga ubone” (loosely translated to: “Plant and you will see!”). A study by Setimela et al. [60] showed that PAN 53 is a highly productive and stable hybrid and this may also explain its consistently high yields as compared to the OPV and to the other hybrids. Despite hybrids being reported to outperform OPVs, our study showed that some hybrids such as MH 30 did not yield more than the OPV in all environments. In fact, there was a cross-over effect at higher yield levels signifying that in such environments it is better to plant OPVs or use better adapted hybrids such as PAN 53. This is in line with the results shown by the biplots which showed that the MH 30 was not as stable as the OPV and yet performed better in almost the same number of environments. Thus, we can partially accept our hypothesis that hybrids yield more than OPVs although this depends on the type of hybrid.

As for the management systems, the CA-based systems consistently outyielded the CPM-L at all yield levels (Fig 8B). This may be attributed to the implementation of the CA principles such as mulch retention. Mulching is crucial in southern African cropping systems especially for moisture retention, soil temperature regulation, and organic matter build-up [22]. Since rainfall is erratic in Malawi as is the case for the rest of southern Africa and limited to only a five-month rainy season, mulching conserved moisture resulting in higher water availability for the plant during in-season dry spells and moderated high temperatures [24], leading to higher yields. The soils in the study area are generally low in organic matter and mulching may have increased the organic matter content of the soil thus improving its properties such as infiltration as was previously shown by Mhlanga et al. [49]. This means that the implementation of CA-based systems is generalizable across the study communities in Malawi and so we accept our third hypothesis that CA-based systems yield more than conventional practices.

### 4.3. Implications of study findings and further research

Despite the findings of this study and preceding work that showed the positive effects of hybrids and CA on productivity, their adoption and use remains hindered by several constraints in the region. Although CA has been reported to increase labour requirements in general mainly due to weed control, the use of the dibble stick on previously formed ridges required less labour and had more economic returns as compared to its use on annually formed ridges [61]. This shows the potential of reduced tillage systems in reducing labour required for land preparation in the long-term. However, a recent study by Thierfelder et al. [56] showed that the integration of more than one crop, which is a component of CA systems, can result in more labour required for planting the surplus crops although with higher economic and nutritional returns. This suggests that extra labour is one of the constraints to the implementation of CA systems. Thus, mechanization becomes relevant in promoting the implementation of CA. Government policies in the region should aim at providing enabling environments for promoting mechanization e.g., by subsidizing or reducing prices for mechanization implements and services [62].

On the other hand, tillage has been used by farmers mainly to control weeds at the beginning of the season and to create a fine seedbed. Thus, during the first years of shifting to minimum soil disturbance, there is a noticeable proliferation of weeds and a shift in weed spectrum which requires farmers to adopt alternative methods of weed control such as the use of herbicides. Yet, besides being environmentally unfriendly, herbicides are either not affordable or inaccessible to the farmers and hence forcing farmers to rely on tillage which is labour intensive. Furthermore, despite decades of agricultural policies that promoted the adoption of hybrid and OPV seed technologies in Malawi, the Seed Act seems to recognize the formal seed system over the farmer’s seed systems and yet farmers’ seed systems will continue to be the most important source of seeds for the majority of smallholder farmers [63]. Yet, common to seed systems of many countries in the region is price collusion among seed suppliers such that seed prices are high and unaffordable to smallholder farmers [64]. Based on this, there is need for the intervention of government and policymakers in regulating input prices and introduction of subsidy programs that improve access to maize hybrid seed and legume by smallholder farmers [65]. Furthermore, the Malawi government may need to start recognizing the farmer seed systems in the Seed Act such that these complement the formal seed system in ensuring that farmers have access to seed at affordable prices.

## 5. Conclusion

In our study we hypothesized that maize yield response in the smallholder communities of Malawi is determined by the G×E×M effect which was not proven by our data. Rather, yield was determined by G×E and E×M effects meaning that maize varietal and management choices are crucial in enhancing productivity under the smallholder farming systems in different environments of Malawi but not necessarily by its three-way interaction. Farmers need to identify the genotypes or management systems that suit their environmental conditions. For example, the use of some maize hybrids such as PAN 53 was shown to increase yield in most of the environments and to be stable across the environments and so farmers may effectively use this hybrid in all environments tested. On the other hand, the CA-based systems also showed to consistently outyield the conventional practice and hence farmers in the study region can adopt these practices for enhanced yield. However, the tested OPV of a similar maturity group showed higher yield stability compared to the hybrid MH 30 and yet was at par in terms of yield performance and so farmers may opt for the OPV over MH 30. This is an important outcome especially considering that OPV seed is less expensive and can be recycled for 3–4 cropping seasons without major yield penalty. The use of CA-based systems either with or without intercrops enhanced yield in environments that received adequate moisture compared to the traditional ridge tillage although with intercrops, extra grain and biomass is produced such that the total system productivity is higher. Overall, we conclude that farmers producing maize in the communities of Malawi should opt for hybrids such as PAN 53 but can go for OPVs where the climate is highly variable, where they have no access to seed or where they are cash constrained. We also recommend that farmers adopt CA-based practices to enhance maize productivity in rural communities of Malawi as this clearly showed benefits over time. Thus, our findings can inform the policy makers in Malawi to maintain programs that promote the use of CA-based farming methods and hybrid seed. This can be achieved through subsidizing land preparation services that are based on CA as well as hybrid seed so that farmers can afford them. Another means would be to increase availability of CA equipment and hybrid seed. We also suggest the development of predictive approaches and simulation modelling as tools that could assist in decision making processes since rainfall and temperature are variable and simulation modelling could predict the effects on cropping systems in a more cost-effective manner. Economic evaluations of the different combinations of CA systems and maize genotypes to understand affordability of the systems by the smallholder farmers is an additional research need and will be addressed in further research.

## Supporting information

S1 Fig Seasonal cumulative rainfall received in the ten communities from 2013/2014 to 2019/2020. The coefficient of variation was calculated for the same period and the error bars denote the standard deviation during the same period. Rainfall was recorded using rain gauges installed at each farmer’s site.
(TIF)

S2 Fig Daily maximum (red lines) and daily minimum (black lines) air temperatures experienced in the four selected communities; a) Chipeni, b) Linga, c) Lemu, and d) Songani; from 2013/2014 to 2019/2020. Temperature data was acquired from the NASA Power website https://power.larc.nasa.gov/data-access-viewer/.
(TIF)

S3 Fig Responses of maize grain yield to rainfall, air temperature, relative humidity, all-sky surface photosynthetic active radiation, wind speed, and root zone soil wetness from 2014 to 2021 across all the environments for Balaka Dowa, Machinga, Nkhotakota, Salima, and Zomba districts. Lines were constructed using second-degree orthogonal regression using retained models. The scatter points represent the raw data. All data for parameters except for rainfall were acquired from the NASA Power website. https://power.larc.nasa.gov/data-access-viewer/.
(TIF)

S1 Table Average values for the weather parameters that were recorded at each site. (DOCX)

S2 Table Polynomial regression results showing the effects of investigated weather parameters on maize grain yield at all sites across all seasons. (DOCX)

S1 Raw data (CSV)
