# Supplementary figures and images for: Understanding the interactions of genotype with environment and management (G×E×M) to enhance maize productivity in Conservation Agriculture systems of Malawi
Source: PLoS One. 2024 Apr 29;19(4):e0298009. doi: 10.1371/journal.pone.0298009 (PMC11057976; doi:10.1371/journal.pone.0298009)

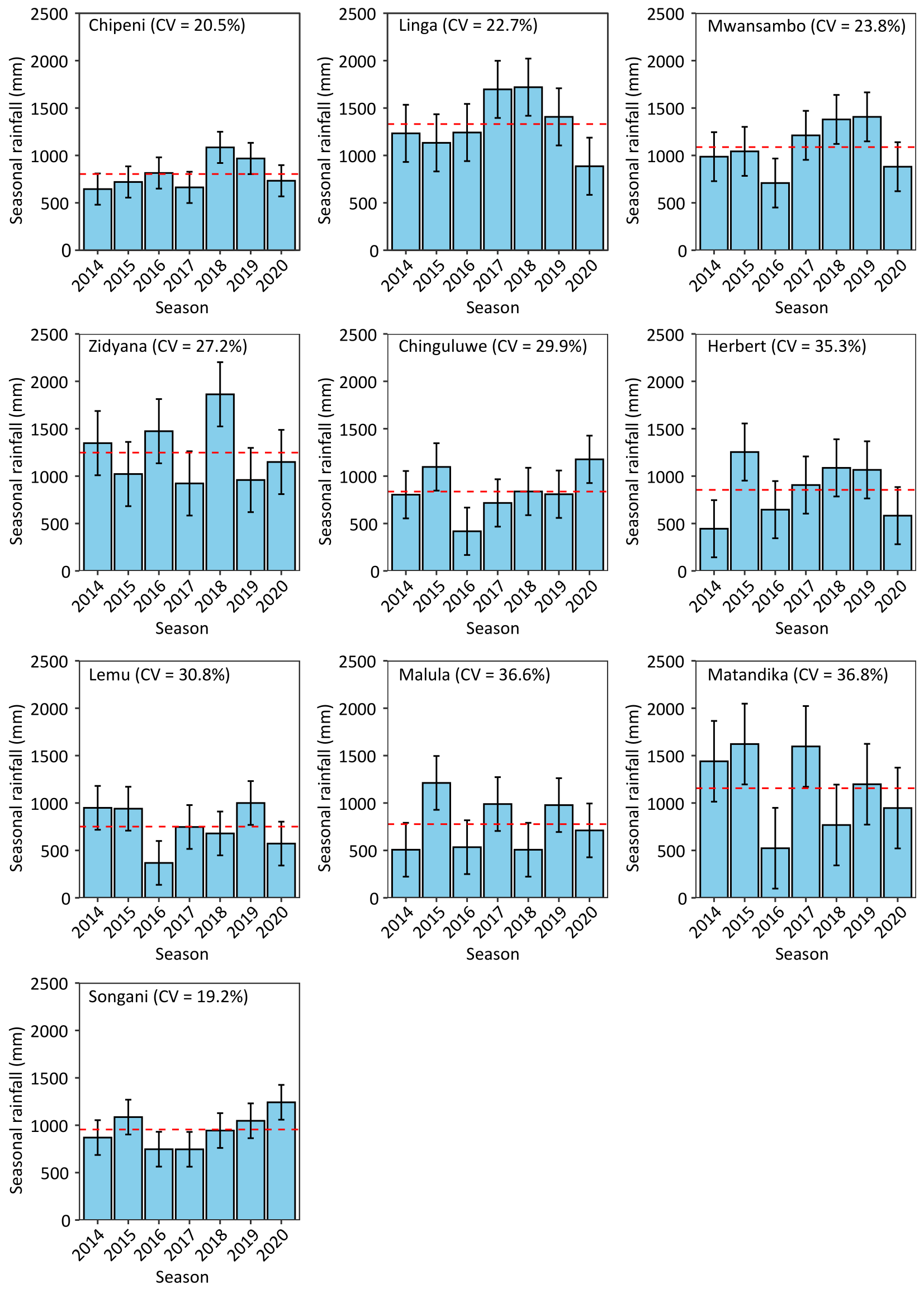

Supplement: S1 Fig — The coefficient of variation was calculated for the same period and the error bars denote the standard deviation during the same period. Rainfall was recorded using rain gauges installed at each farmer’s site. (TIF) [file pone.0298009.s001.tif]

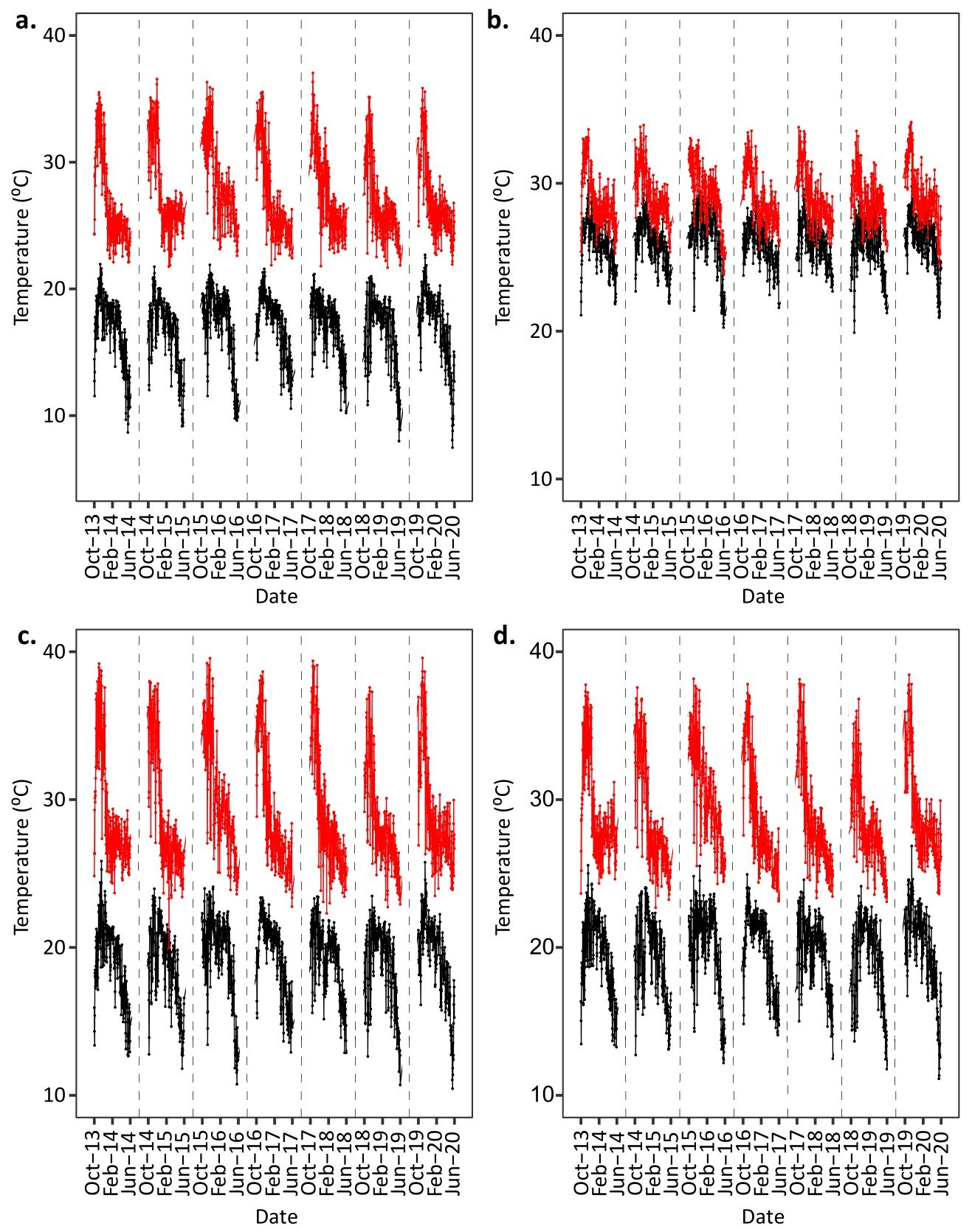

Supplement: S2 Fig — Daily maximum (red lines) and daily minimum (black lines) air temperatures experienced in the four selected communities; a) Chipeni, b) Linga, c) Lemu, and d) Songani; from 2013/2014 to 2019/2020. Temperature data was acquired from the NASA Power website https://power.larc.nasa.gov/data-access-viewer/. (TIF) [file pone.0298009.s002.tif]

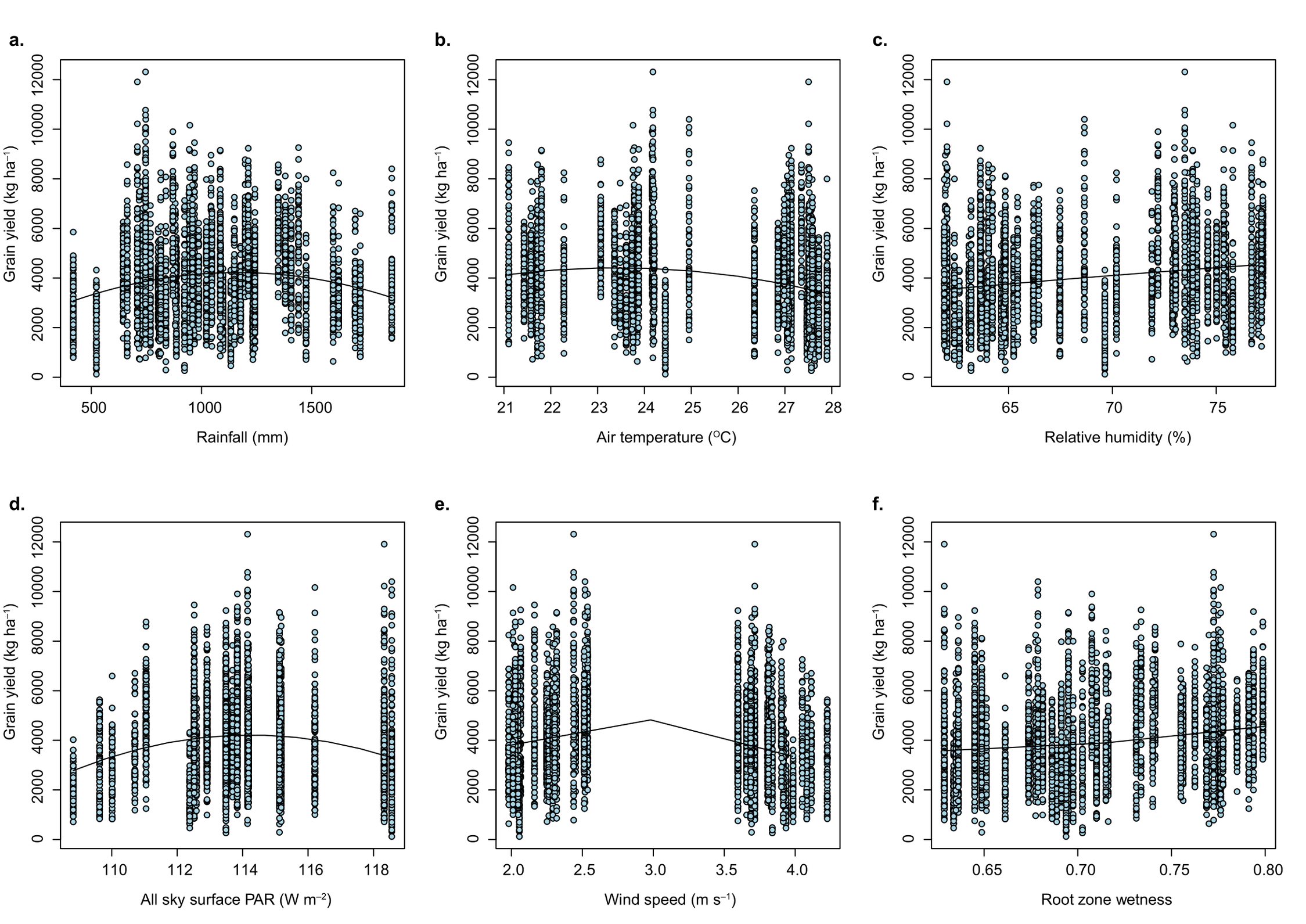

Supplement: S3 Fig — Lines were constructed using second-degree orthogonal regression using retained models. The scatter points represent the raw data. All data for parameters except for rainfall were acquired from the NASA Power website. https://power.larc.nasa.gov/data-access-viewer/. (TIF) [file pone.0298009.s003.tif]
