# Supplementary material for: Understanding the interactions of genotype with environment and management (G×E×M) to enhance maize productivity in Conservation Agriculture systems of Malawi
Source: PLoS One. 2024 Apr 29;19(4):e0298009. doi: 10.1371/journal.pone.0298009 (PMC11057976; doi:10.1371/journal.pone.0298009)
Supplement: S1 Table — (DOCX) [file pone.0298009.s004.docx]

| **S1 Table. Average values for the weather parameters that were recorded at each site** | | | | |
| --- | --- | --- | --- | --- |
|  | Weather parameter† | | | |
| Site | Relative humidity | PAR | Wind speed | Root zone soil wetness |
| Chinguluwe | 64.3 | 114.4 | 3.7 | 0.7 |
| Chipeni | 74.1 | 114.5 | 2.3 | 0.7 |
| Linga | 63.9 | 110.7 | 4.1 | 0.7 |
| Matandika | 74.0 | 114.5 | 2.0 | 0.8 |
| Mwansambo | 64.4 | 114.5 | 3.7 | 0.7 |
| Songani | 73.5 | 114.5 | 2.5 | 0.8 |
| Zidyana | 63.9 | 114.5 | 3.9 | 0.7 |
| Mean | 68.3 | 114.0 | 3.2 | 0.7 |
| Coefficient of variation | 7.6 | 1.3 | 27.2 | 6.6 |
| †Weather parameters were obtained from the from the NASA Prediction of Worldwide Energy Resource (POWER) database (https://power.larc.nasa.gov/data-access-viewer/). Wind speed = wind speed at 2m height above the soil surface; PAR = all-sky surface photosynthetic active radiation | | | | |
