# Supplementary material for: Understanding the interactions of genotype with environment and management (G×E×M) to enhance maize productivity in Conservation Agriculture systems of Malawi
Source: PLoS One. 2024 Apr 29;19(4):e0298009. doi: 10.1371/journal.pone.0298009 (PMC11057976; doi:10.1371/journal.pone.0298009)
Supplement: S2 Table — (DOCX) [file pone.0298009.s005.docx]

| **S2 Table. Polynomial regression results showing the effects of investigated weather parameters on maize grain yield at all sites across all seasons** | | | | |
| --- | --- | --- | --- | --- |
| Source† | Estimate | Standard error | t- value | Pr(>\|t\|)¶ |
| (Intercept) | 3974.93 | 28.02 | 141.875 | < 2e-16*** |
| poly(Rainfall, 2)1 | 13447.7 | 1967.51 | 6.835 | 9.65e-12*** |
| poly(Rainfall, 2)2 | -12687.56 | 1899.69 | -6.679 | 2.79e-11*** |
| poly(Air temperature, 2)1 | 77652.4 | 14814 | 5.242 | 1.68e-07*** |
| poly(Air temperature, 2)2 | 32343.47 | 5500.84 | 5.88 | 4.50e-09*** |
| poly(RH, 2)1 | 40070.05 | 8905.33 | 4.5 | 7.03e-06*** |
| poly(RH, 2)2 | -15107.64 | 3102.49 | -4.87 | 1.17e-06*** |
| poly(PAR, 2)1 | -2212.39 | 2510.13 | -0.881 | 0.3782^NS^ |
| poly(PAR, 2)2 | -2506.78 | 1932.88 | -1.297 | 0.1947^NS^ |
| poly(Wind speed, 2)1 | -59999.8 | 11982.96 | -5.007 | 5.80e-07*** |
| poly(Wind speed, 2)2 | -35713.87 | 2866.21 | -12.46 | <2e-16*** |
| poly(RZW, 2)1 | 6800.81 | 3683.59 | 1.846 | 0.0649. |
| poly(RZW, 2)2 | 5683.84 | 2421.71 | 2.347 | 0.0190* |
| †RH = relative humidity; PAR = all sky surface photosynthetic active radiation; RZW = root zone wetness. ¶*P*-values followed by asterisks show significant differences at * and *** representing P < 0.001 and P < 0.05, and NS means not significant. | | | | |
